# Transoral endoscopic thyroidectomy submental vestibular approach for early-stage papillary thyroid carcinoma: a systematic review and meta-analysis

**DOI:** 10.1007/s00423-024-03377-x

**Published:** 2024-07-04

**Authors:** Mahmoud Diaa Hindawi, Ahmed Hamdy, G. Ali, Ruaa Mustafa Qafesha, Wesam Soliman, Haitham Salem, Eslam Bali, Amr Elrosasy

**Affiliations:** 1 Faculty of Medicine, Al-Azhar University, Cairo, Egypt; 2 Faculty of Medicine, Ogarev Mordovia State University, Saransk, Russia; 3 Faculty of Medicine, Al-Quds University, Jerusalem, Palestine; 4 Faculty of Medicine, Assiut University, Assiut, Egypt; 5 Faculty of medicine, Ain shams University, Cairo, Egypt; 6 Faculty of Medicine, Cairo University, Cairo, Egypt

**Keywords:** Papillary thyroid carcinoma, Endoscopic thyroidectomy, Transoral endoscopic thyroidectomy submental vestibular approach, Transoral endoscopic thyroidectomy vestibular approach, Conventional open thyroidectomy

## Abstract

**Purpose:**

Our study aimed to compare the effectiveness and complications of the transoral endoscopic thyroidectomy submental vestibular approach (TOETSMVA) versus the transoral endoscopic thyroidectomy vestibular approach (TOETVA) or conventional open thyroidectomy (COT) in patients with early-stage papillary thyroid carcinoma (PTC).

**Methods:**

We searched online databases up to January 2024. The outcomes were analyzed using RevMan 5.4 and inverse variance.

**Results:**

Seven studies (two RCTs and five retrospective cohort studies) were included. We established higher significance differences for TOETSMVA in comparison with TOETVA in terms of all primary outcomes; operation time, hospital stay, number of resected lymph nodes [MD -21.05, 95% CI= -30.98, -11.12; *p* < 0.0001], [MD -1.76, 95% CI= -2.21, -1.32, *p* < 0.00001], [MD -2.99, 95% CI= -19.75, 13.76, *p* < 0.73], [MD -0.83, 95% CI = -1.19 to -0.47; *p* < 0.00001], respectively, except the drainage volume, it showed no difference [MD -2.99, 95% CI= -19.75, 13.76, *p* < 0.73]. In secondary outcomes, it was favored only in mandibular numbness and return to normal diet outcomes. Additionally, TOETSMVA compared with COT showed a significant difference in drainage volume, pain, cosmetic effect, and satisfaction score.

**Conclusions:**

TOETSMVA showed a significant improvement compared to the TOETVA in operation time, hospital stay, number of resected lymph nodes, mandibular numbness, and return to normal diet but did not show a difference in drainage volume. However, TOETSMVA was better in cosmetic effect, drainage volume, satisfaction, and pain scores compared with COT. Further RCTs with larger sample size, multicentral, and longer follow-up are necessary to evaluate the limitations.

**Supplementary Information:**

The online version contains supplementary material available at 10.1007/s00423-024-03377-x.

## Introduction

Thyroidectomy is indicated for various benign and malignant conditions including symptomatic goiter, differentiated cancer, metastatic cancer in the thyroid gland, and hyperthyroidism refractory to medical treatment [1]. Among the differentiated neoplasms, papillary and follicular thyroid carcinoma approximates about 97% of neoplasms with high survival and favorable outcomes [2–4]. It is caused by a genetic mutation of mitogen-activated protein kinase leads to activation and malignant transformation [5]. Patients may be asymptomatic in the early stages and then progress to dysphagia and dysphonia up to respiratory distress with late stages [6]. Papillary carcinoma can metastasize in cervical lymph nodes hindering effective treatment and impacting the patient’s survival [7].

As surgical treatment is the preferred method for removing malignant thyroid tumors, many studies have been conducted over a long time to find the best patient care. Hence, over the past 150 years, operative thyroidectomy has been updated and various techniques have been developed. Also, as conventional open thyroidectomy (COT) has a psychological impact on patients and affects the quality of life by leaving a 10 cm scar [8], surgeons tend to head forward to minimally invasive procedures for better aesthetic appearance as long as the same effect of the conventional thyroidectomy [9, 10].

Transluminal endoscopic surgery has attracted the interest of a wide range of surgeons [11]. The first endoscopic right thyroid lobectomy was thought to be feasible with an interesting cosmetic effect [12]. Currently, the transoral endoscopic thyroidectomy vestibular approach (TOETVA) is the scarless option and the most popular approach which achieves a good therapeutic effect and reducing the discomfort of the patients with a good cosmetic effect [13–15]. However, the anatomic obstacles that may develop cutaneous paralysis of the midline chin tend surgeons for a better solution [16].

Recently, the transoral endoscopic thyroidectomy submental vestibular approach (TOETSMVA) has been reported to be more favorable in patients with papillary and follicular thyroid carcinoma in all grades of surgical complications [17–20]. Also, this approach does not impact on costs and not require dedicated endoscopic or robotic instruments [18]. The current literature reports vary between TOETSMVA, TOETVA, and COT and a meta-analysis is mandatory to evaluate these reports. Our systematic review and meta-analysis (SR&MA) aims to strictly compare all the complications and surgical outcomes between TOETSMVA, TOETVA, and COT, providing valuable information and insight for professional healthcare providers and researchers in the field.

## Methods

We reported our work in accordance with the Preferred Reporting Items of Systematic Reviews and Meta-Analysis (PRISMA statement) guidelines [21]. We used the Cochrane Handbook of Systematic Reviews of Interventions as guidance [22]. Prospectively this study was registered on PROSPERO (CRD42024510260).

### Eligibility criteria

In this SR&MA, the studies met the inclusion criteria if: (1) their population had early-stage papillary thyroid carcinoma, aged over 18 years old; (2) the intervention group is TOETSMVA; (3) the control group is TOETVA or COT; (4) randomized controlled trials or cohort (retrospective, prospective) studies; and (5) studies at least reporting one of our primary efficacy and complication outcomes.

We excluded (1) animal studies; (2) case series/case reports studies; (3) thesis; (4) conference abstracts and (5) all single-arm studies.

### Search strategy and data collection process

We searched Medline via PubMed, Scopus, Ovoid, Web of Science, China Knowledge Network (CNKI), and Cochrane Central for possible studies without language restriction in Jan 2024. We used MESH keywords for a sensitive search strategy. The details of each database search strategy are in the (Electronic supplementary material (ESM) 1).

The results were divided into three sections. Using Rayyan [23], two authors independently screened each section in two steps: initially title/abstract screening then carefully full text for eligible studies. Also, the reference to the new techniques was strictly reviewed manually to enhance the productivity of the study. The same was for data extraction, two authors extracted the data independently for each section using Google Docs. The final review was conducted using an online meeting and any conflict was solved by discussion.

### Data items

We comprehensively included all the possible outcomes from our included studies for the analysis. The Primary outcomes were operation time, length of hospital stay, postoperative drainage volume, and the number of resected lymph nodes. Secondary outcomes were pain (VAS score), cosmetic effect, temporary hoarseness, lower lip numbness, drinking or cough incidence, recurrent laryngeal nerve injury, mandibular numbness, intraoperative blood loss, days to return to normal diet, and satisfaction score. Each outcome was defined by included studies [19, 20], [24–28].

### Quality assessment

We used the Cochrane risk-of-bias tool (ROB 2) for the included RCTs [29], which has six domains: (1) randomization process, (2) deviation from intended intervention, (3) missing outcome data, (4) measuring outcome, (5) selection of the reported, outcome and (6) other bias. At every stage of each domain, the authors had to decide (yes, no, probably yes, probably no, or no information). Studies were required to assign grades of (1) low risk, (2) moderate concerns, and (3) high risk, according to ROB 2. We used the Newcastle Ottawa scale (NOS) to assess the quality of the cohort studies. NOS has 3 main domains for assessment: (1) SELECTION (2) COMPARABILITY and (3) OUTCOME. Authors had to give a point for each arm in every domain. Studies were required to assign grades of (1) good quality, (2) fair quality, and (3) poor quality, according to NOS. We used the Grading of Recommendations Assessment, Development, and Evaluation (GRADE) guidelines to evaluate the degree of evidence. Any conflicts were resolved by a third author [30, 31].

### Statistical analysis

Revman 5.4 and OpenMeta analyst [32]were used for the study analysis. Mean difference (MD) and standard deviation (SD) were used to describe the continuous variables. While for categorical variables, we used Risk ratio (RR) and 95% CI. A *p* value of < 0.05 indicated statistical significance. Heterogeneity was assessed by Chi-square (I^2^) test (Cochrane Q test) and *p* < 0.1 was considered significant. I^2^ test (0–20%) = may not be significant, (30–60%) = maybe represent moderate heterogeneity, (50–90%) = may represent substantial heterogeneity, and (75–100%) considerable heterogeneity, (Cochrane Handbook, Part 2, Chap. 9) [33]. Heterogeneity between the included studies was solved by using a random effect model instead of a fixed effect, and sensitivity analysis. We conducted subgroup analysis according to different types of interventions; TOETSMVA VS TOETVA OR COT.

### Reporting bias assessment

We sought to apply a publication bias assessment using the funnel plot method, but according to Egger *el al.* [34], there were not enough included studies, the assessment was not feasible statistically as it requires at least ten studies [34].

## Results

### Study selection and characteristics

**Fig. 1:**
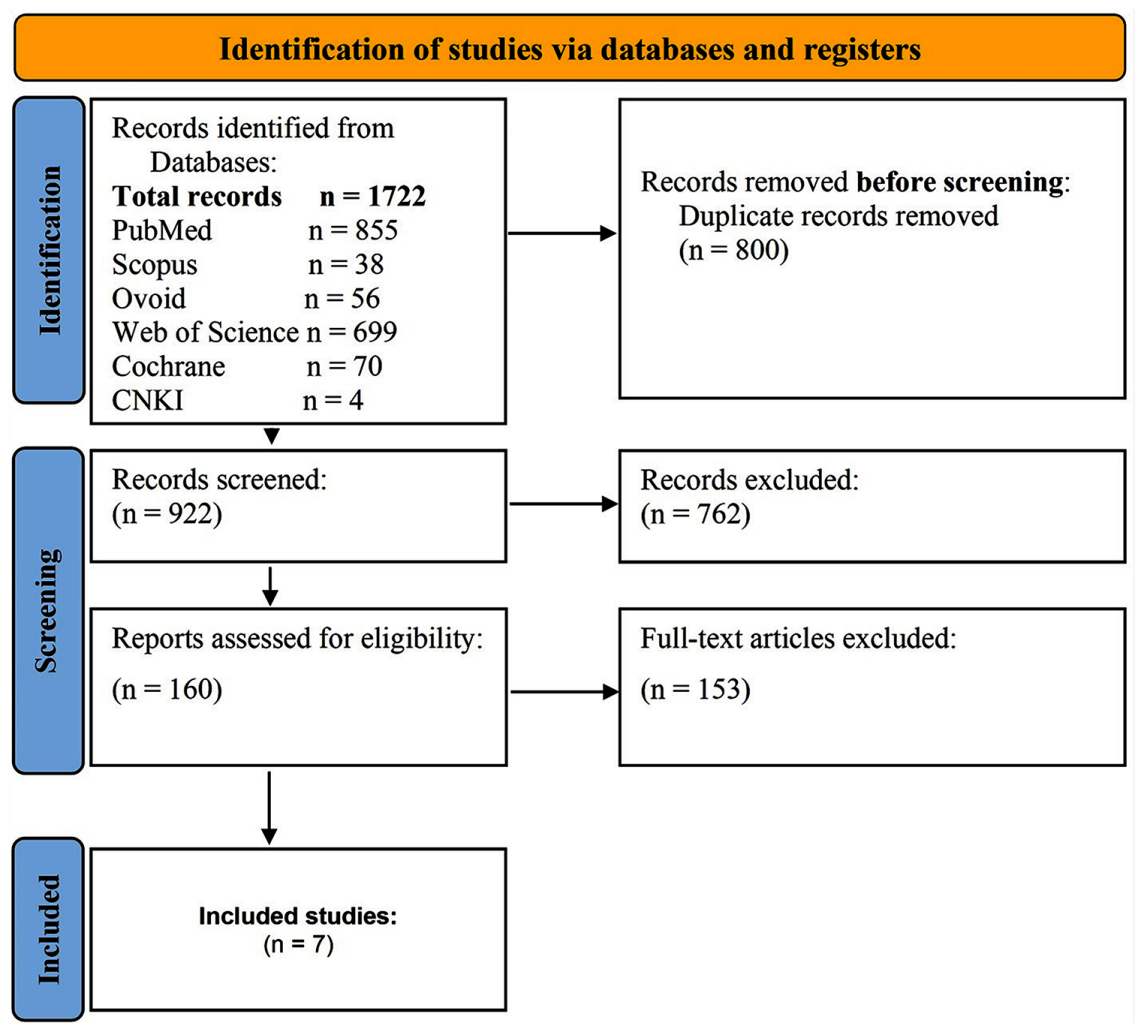
PRISMA flow diagram of the study selection process

A comprehensive search of the electronic databases yielded 1722 studies. After removing duplicates, a total of 922 articles were included in the title and abstract screening. Ultimately, seven studies (two RCTs and five retrospective cohort studies) met the criteria for inclusion in the analysis. For a detailed description of the selection process, please refer to (Fig. 1). The seven studies collectively involved 636 patients aged 38–43 years, with a predominance of females. All studies included in the analysis were published within a timeframe of two years (2022–2023). Four of them compared TOETSMVA to the TOETVA procedure, and the other three studies compared TOETSMVA to open surgery. The summary and characteristics of the included studies are summarized in Table 1 and 2 respectively.

**Table 1:** Summary of the included studies

Studies	Study design	Country	Sample	Follow-up duration	Surgical technique	Conclusion
Zanbin 2023 [25]	RCT	China	100	2 years	**TOETSMVA** **Vs** **TOETVA**	TOETSMVA in PTC patients showed improvements in perioperative indicators, reduced incidences of lower lip numbness and postoperative pain, and enhanced the quality of life.
Chi 2022 [26]	retrospective cohort	China	60	8 months		TOETSMVA, a new approach, helped extend and shorten both surgical and hospitalization times. It alleviates postoperative pain and discomfort with a highly cosmetic effect
Ma 2023 [20]	Retrospective Cohort	China	156	6 months		Although, TOETSMVA has the same surgical effectiveness as traditional TOETVA, it can reduce the probability of mandibular numbness and improve perioperative life quality in early-stage PTC patients.
Teng 2022 [19]	Retrospective Cohort	China	63	6 months		Both approaches, TOETSMVA and TOETVA, demonstrate similar efficacy in treating early PTC. However, the TOETSMVA was associated with a reduction in operation time and an improvement in the quality of life for patients.
Chen 2023 [28]	retrospective cohort	China	112	3 months	**TOETSMVA** **Vs** **COT**	TOETSMVA showed significant efficacy and safety profile, same as COT. However, it was significantly higher in terms of the cosmetic effect.
Li 2023 [24]	RCT	China	90	3 months		In comparison with COT, TOETSMVA showed good clinical efficacy, caused less pain, had a good cosmetic effect.
Zhi-qiang 2022 [27]	retrospective cohort	China	103	6 months		For PTC patients, TOETSMVA was not inferior to COT in terms of safety outcomes and is suitable for patients who have a higher concern for cosmetic impact.

**Table 2:** Baseline characteristics for the population of the included studies

Study ID	Age, mean (SD), y		Sex (female), *N* (%)		BMI, mean (SD)		Tumor size, mean (SD)		Tumor diameter mean (SD)		Tumor location *N* (%)			
											(left)		(Right)	
	Intervention	Comparator	Intervention	Comparator	Intervention	Comparator	Intervention	Comparator	Intervention	Comparator	Intervention	Comparator	Intervention	Comparator
Zanbin 2023 [25]	41.43 (5.28)	41.38 (5.25)	37 (74)	76 (24)	N/A	N/A	N/A	N/A	N/A	N/A	30 (60)	29 (58)	20 (40)	21 (42)
Chi 2022 [26]	32.30 (6.92)	31.50 (9.15)	27 (90)	29 (97)	N/A	N/A	N/A	N/A	0.77 (0.46)	0.61 (0.22)	N/A	N/A	N/A	N/A
Zhi-qiang 2022 [27]	36.43 (8.54)	39.74 (7.48)	20 (74.07)	31 (40.79)	N/A	N/A	N/A	N/A	1.43 (0. 68)	1.65 (0.84)	15 (55.55)	41 (53.95)	12 (44.44)	35 (46.05)
Chen 2023 [28]	34.97 (60.34)	33.98 (43.8)	60 (100)	52 (100)	22.8 (15.72)	21.39 (14.35)	N/A	N/A	0.69 (2.01)	0.65 (2.16)	36 (60.00)	28 (53.85)	24 (40.00)	24 (46.15)
Li 2023 [24]	40.16 (10.28)	43.27 (11.09)	33 (73.33)	35 (77.78)	N/A	N/A	9.42 (5.91)	9.76 (5.82)	N/A	N/A	24 (53.33)	16 (35.56)	12 (26.67)	21 (46.67)
Ma 2023 [20]	30.64 (6.97)	28.58 (7.81)	31 (73.8)	102 (89.4)	26.94 (2.59)	25.70 (3.52)	10.57 (5.18)	8.92 (4.19)	N/A	N/A	N/A	N/A	N/A	N/A
Teng 2022 [19]	32.7 (6.2)	30.4 (8.2)	26 (96)	33 (91)	26.73 (2.96)	25.70 (3.11)	N/A	N/A	9.74 (4.65)	10.78 (4.64)	N/A	N/A	N/A	N/A

All **continuous** variables (Age, BMI, Tumor size, Tumor diameter) are described as Mean± (SD), and all **categorical** variables (gender: Female, Tumor location) are described as N (%); Not Applicable (N/A), Number (N), Standard deviation (SD

### Quality of the included studies

**Fig. 2:**
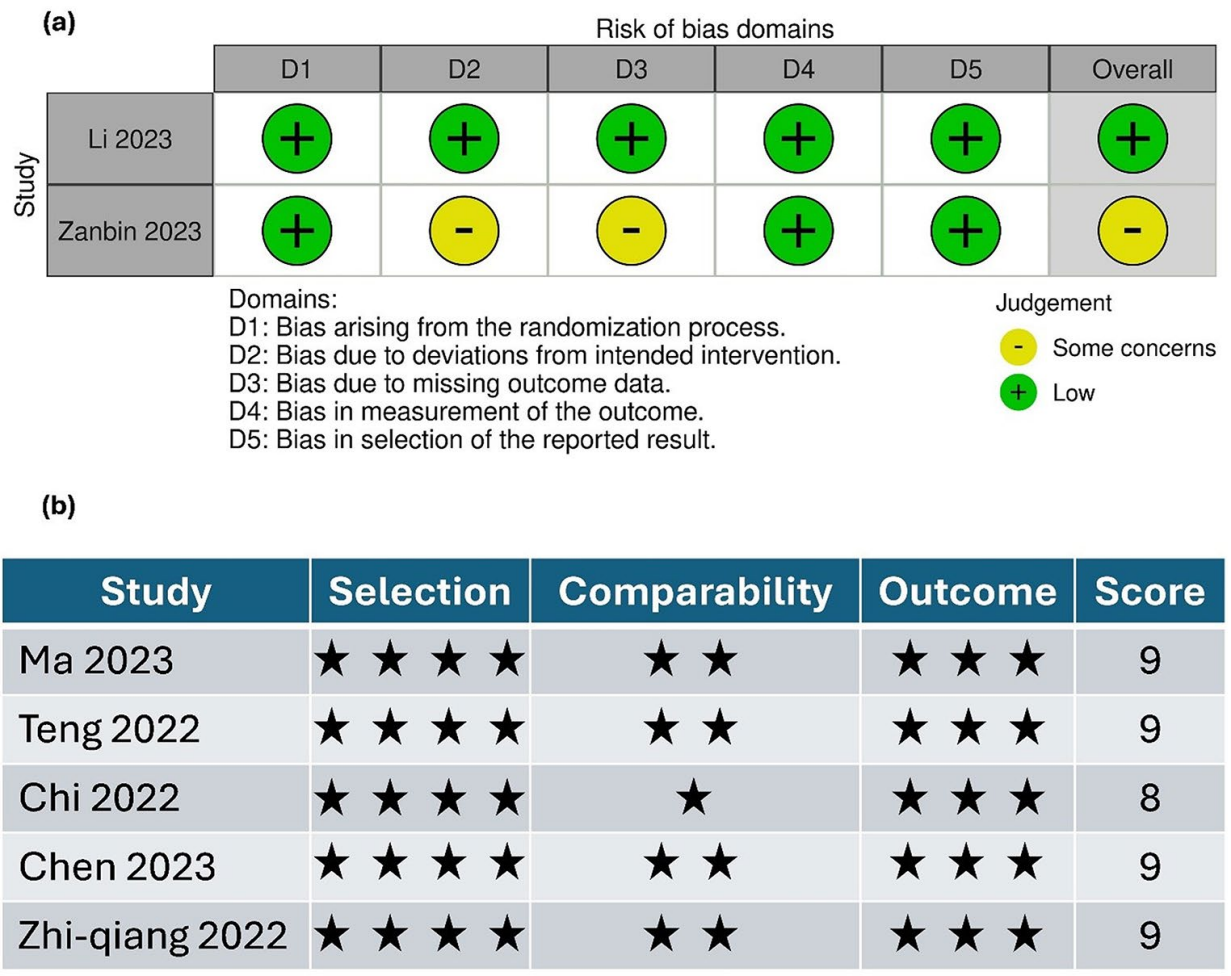
**(a)** Risk of bias summary for randomized controlled trials using ROB2. **(b)** summary of Newcastle-Ottawa Scale (NOS) quality assessments

The risk of bias was assessed using the ROB 2 tool for the two RCTs. One trial exhibited an overall low risk of bias. However, another study was deemed to raise concerns regarding bias related to missing data outcomes and deviations from the intended intervention. The risk of bias summary for randomized controlled trials using ROB-2 is shown in (Fig. 2. a) and the risk of bias graph is presented in (ESM. 2). For the five other retrospective studies, NOS quality assessments indicated high quality, with nine points for four studies and eight for one study as shown in (Fig. 2 . b). The certainty of evidence is shown in a GRADE table (ESM. 9).

## Primary outcomes

### Operation time

**Fig. 3:**
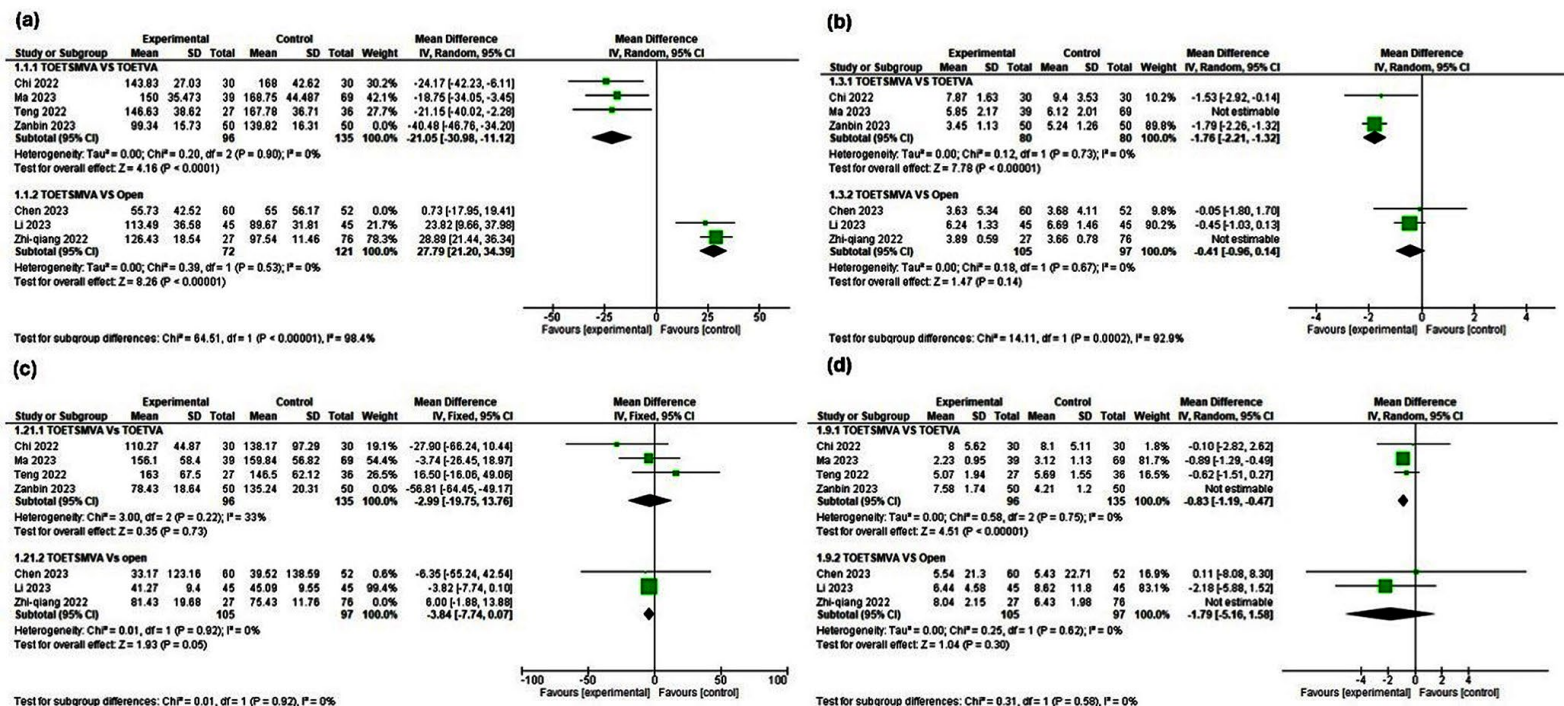
Meta-analysis forest plot containing the primary outcomes **(a)** operation time, **(b)** length of hospital stay, **(c)** drainage volume, and **(d)** number of lymph nodes resected

All the included studies reported this outcome. TOETSMVA was compared with the TOETVA in four studies, and the results indicated significantly shorter operation time with the TOETSMVA [MD -27.7, 95% CI = -40.78, -14.62, *p =* 0.0001]. The analysis model showed substantial heterogeneity, which was resolved by conducting a sensitivity analysis excluding the study of Zanbin et al. [25]. After sensitivity analysis, the operation time still significantly shorter with the TOETSMVA [MD -21.05, 95% CI= -30.98, -11.12; *p* < 0.0001], as shown in (Fig. 3. a).

Three other studies compared the TOETSMVA approach to COT, the results indicated a significantly shorter operation time in the open surgery group than in the TOETSMVA group [MD 19.83, 95% CI = 5.21, 34.44, *p* < 0.008]. The analysis model showed substantial heterogeneity and was resolved using sensitivity analysis by removing the study of Chen et al. [28]. Also, after sensitivity analysis, the results still showed a significantly shorter operation time in the COT group than in the TOETSMVA group [MD 27.79, 95% CI = 21.20, 34.39, *p* < 0.00001], as shown in (Fig. 3. a).

### Hospital stay

Out of the seven studies analyzed, six studies provided data on this outcome. Three studies of the six conducted a comparative analysis between the TOETSMVA and TOETVA surgery groups, demonstrating a significantly shorter hospital stay in the TOETSMVA group, despite substantial heterogeneity [MD -1.20, 95% CI= -2.27, -0.13, *p* < 0.03]. Upon removing the study of Ma et al. [20], the heterogeneity was resolved, and the results continued to support TOETSMVA, showing a significantly shorter hospital stay [MD -1.76, 95% CI= -2.21, -1.32, *p* < 0.00001], as illustrated in (Fig. 3. b).

The remaining three studies compared TOETSMVA to COT, indicating no significant difference in hospital stay [MD -0.04, 95% CI= -0.57, -0.49, *p* < 0.87]. Moderate heterogeneity was detected and resolved by excluding data from the study of Zhi-qiang et al. [27]. Following sensitivity analysis, the results demonstrated no significant difference between TOETSMVA and COT [MD -0.41, 95% CI= -0.96, 0.14, *p* < 0.14], as shown in (Fig. 3. b).

### Drainage volume

Seven studies provided data on this outcome. Among these, four studies compared drainage volume between TOETSMVA and TOETVA, revealing significant difference for TOETSMVA [MD -47.54, 95% CI= -54.49, -40.49, *p* < 0.00001] but heterogeneity has been considered. It was solved by sensitivity analysis by excluding the study of Zanbin et al. [25]. After that, the result did not favor any group [MD -2.99, 95% CI= -19.75, 13.76, *p* < 0.73], as illustrated in (Fig. 3. c).

The remaining three studies compared TOETSMVA with the COT. The results did not show a preference for either group [MD -1.90, 95% CI= -5.40, 1.60; *p* = 0.29] but there was significant substantial heterogeneity. Using sensitivity analysis by excluding the study Zhi-qiang et al. [27] to address heterogeneity, the results indicated a significant difference for TOETSMVA [MD -3.84, 95% CI= -7.74, 0.07, *p* = 0.05], as demonstrated in (Fig. 3. c).

### Number of resected lymph nodes

Data for this outcome was obtained from seven studies. Among these, four studies compared the TOETSMVA and TOETVA groups, showing no significant difference between the groups [MD 0.47, 95% CI = -2.06 to -3.01, *p* < 0.71]. However, significant heterogeneity among studies was observed. This was addressed by excluding the study of Zanbin et al. [25] from the analysis. Following this, it was noted that the TOETVA group had a significantly higher number of resected lymph nodes [MD -0.83, 95% CI = -1.19 to -0.47; *p* < 0.00001], as demonstrated in (Fig. 3. d).

The remaining three studies compare TOETSMVA with COT. The meta-analysis revealed no significant difference between the two procedures [MD 0.34, 95% CI= -2.39, 3.07; *p* < 0.81]. Moderate heterogeneity among studies was observed and resolved by excluding Zhi-qiang et al. [27]. However, it still showed no statistical difference between all groups [MD -1.79, 95% CI= -5.16, 1.58; *p* < 0.30], as shown in (Fig. 3. d).

## Secondary outcomes

### Pain (VAS score)

**Fig. 4:**
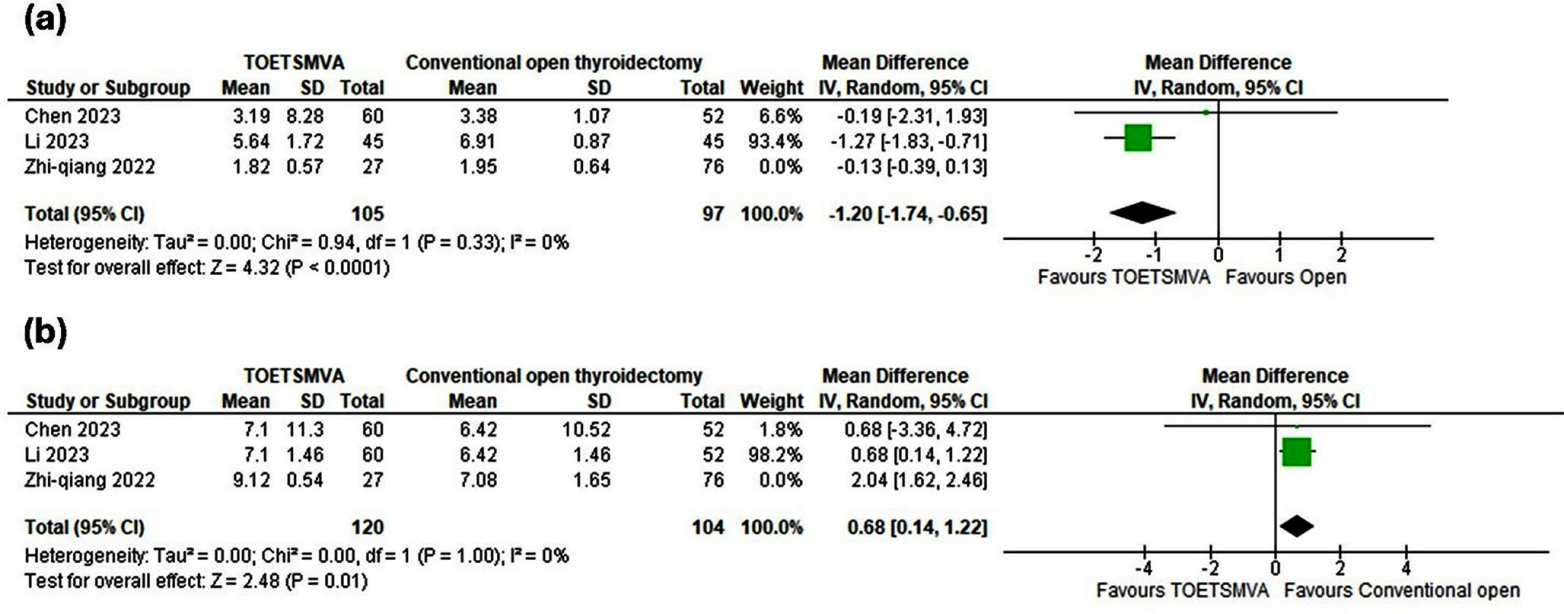
Meta-analysis forest plots containing **(a)** pain (VAS score), **(b)** cosmetic effect

Three studies reported pain, calculated by VAS score, compared TOETSMVA with the COT. The analysis revealed no statistically significant difference [MD -0.60, 95% CI= -1.56, 0.36; *p* = 0.22]. A considerable heterogeneity was evaluated and then we resolved it by using sensitivity analysis and removing the study of Zhi-qiang et al. [27]. After sensitivity analysis, the result was statistically significant for TOETSMVA [MD -1.20, 95% CI= -1.74, -0.65; *p* > 0.0001], as shown in (Fig. 4. a).

### Cosmetic effect

Three studies compared TOETSMVA with COT. The results described a statistically significant difference for TOETSMVA. A considerable heterogeneity was evaluated and then we resolved it by using sensitivity analysis excluding Zhi-qiang et al. [27]. After sensitivity analysis, the result was statistically significant for TOETSMVA [MD 0.68, 95% CI = 0.14, 1.22; *p* = 0.01], as shown in (Fig. 4. b).

### Temporary hoarseness

**Fig. 5:**
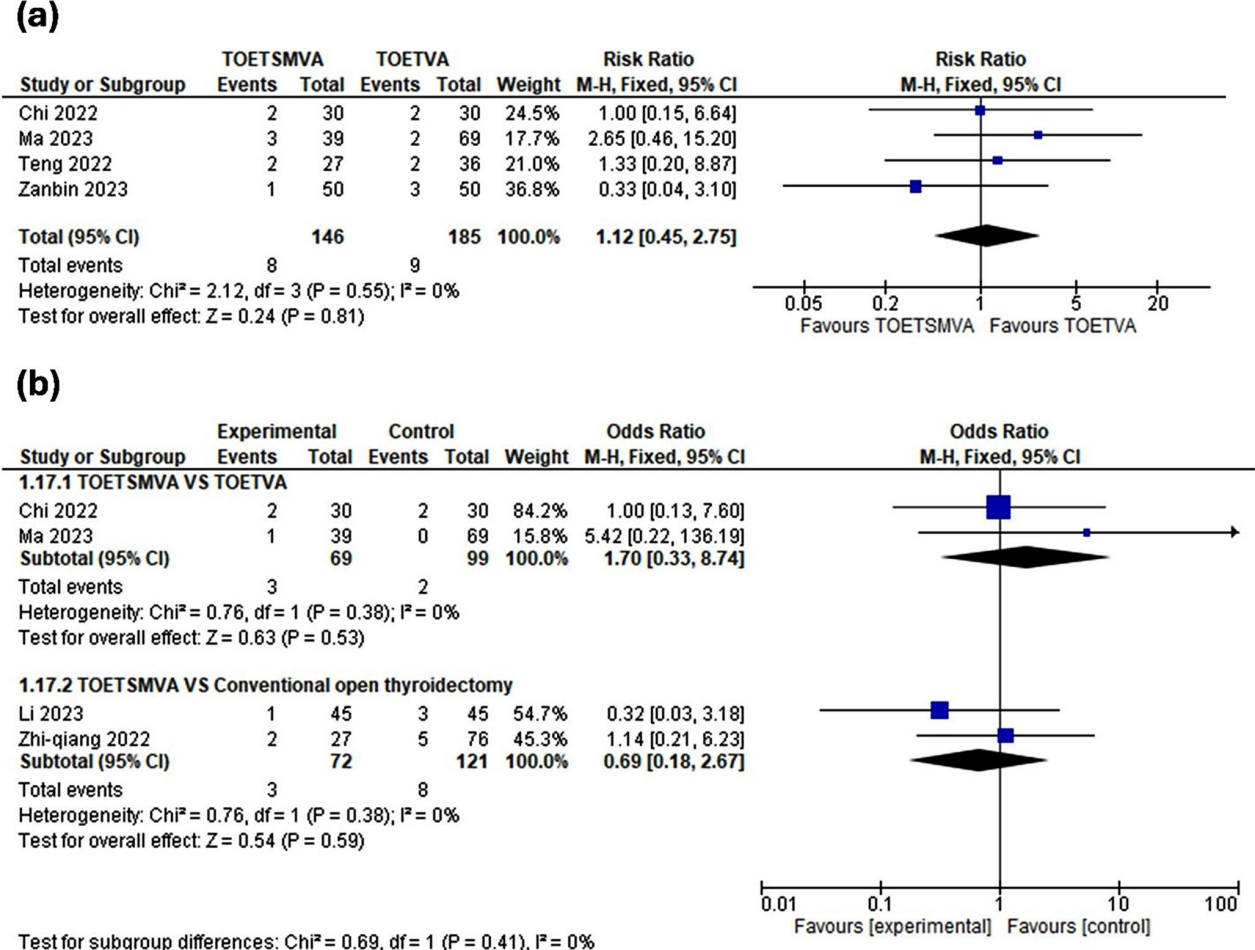
Meta-analysis forest plots containing **(a)** temporary hoarseness, and **(b)** recurrent laryngeal nerve injury

Four studies compared TOETSMVA with TOETVA. The studies were homogenous and meta-analysis revealed no statistically significant difference [RR 1.12, 95% CI = 0.45, 0.75; *p* = 0.81], as shown in (Fig. 5. a).

### Recurrent laryngeal nerve injury

Two studies compared TOETSMVA with TOETVA. The studies were homogenous and meta-analysis revealed no statistically significant difference [OR 1.70, 95% CI = 0.33, 8.74; *p* = 0.53], as shown in (Fig. 5. b).

Two studies compared TOETSMVA with COT. The result was homogenous and revealed no difference [OR 0.69, 95% CI = 0.18, 2.67; *p* = 0.59], as shown in (Fig. 5. b).

### Drinking or cough incidence

Four studies compared TOETSMVA with TOETVA. The result was homogenous and revealed no difference [OR 0.71, 95% CI = 0.20, 2.49; *p* = 0.83], as shown in (ESM. 3).

### Mandibular numbness

Two studies compared TOETSMVA with TOETVA. The result was homogenous and revealed a statistically significant difference [OR 0.23, 95% CI = 0.06, 0.90; *p* = 0.03], as shown in ESM. 4.

### Intraoperative blood loss

Two studies compared TOETSMVA with COT. The analysis was homogenous, and its result revealed no difference [MD 2.42, 95% CI= -2.76, 7.60; *p* = 0.36], as shown in ESM. 5.

### Return to normal diet

Two studies compared TOETSMVA with TOETVA. The analysis was homogenous, and there was a statistically significant difference for the TOETSMVA [MD -0.64, 95% CI= -0.95, -0.34; *p* = 0.0001], as shown in ESM. 6.

### Satisfaction score

Two studies compared TOETSMVA with COT. The analysis was homogenous, and there was a statistically significant difference for the TOETSMVA [MD 0.90, 95% CI = 0.61, 1.20; *p* = 0.00001], as shown in ESM. 7.

### Lower lip numbness

Three studies compared TOETSMVA with TOETVA. The studies were homogenous and meta-analysis revealed no statistically significant difference [OR 0.28, 95% CI = 0.08, 0.94; *p* = 0.59], as shown in ESM. 8.

## Discussion

Previous SR&MA were published in the topic of endoscopic thyroid surgery as a minimally invasive surgery. But the main limitation is that they compared the whole endoscopic thyroid surgery over the COT [35, 36]. Recently A comprehensive network meta-analysis described the surgical outcomes of all different endoscopic thyroidectomies alone such as gasless unilateral transaxillary approach, bilateral axillo-breast approach, axillo-bilateral breast approach, unilateral axillo-breast approach, chest-breast approach, anterior chest approach, TOETVA and COT [37]. They conclude that each different approach carries its advantages and the selection between them is based on the patient’s selection. Our meta-analysis reports a novel technique TOETSMVA in comparison with TOETVA and COT. TOETSMVA was statistically better than TOETVA in terms of (operation time, hospital stay, the number of lymph nodes resected, mandibular numbness, and return to normal diet) but was not statistically better than TOETVA in terms of (drainage volume, temporary hoarseness, drinking or cough incidence, and recurrent laryngeal nerve injury, and lower lip numbness). Our results were consistent with the results of Chi et al. [26], Ma el al. [20] Teng el al. [19], and Zanbin et al. [25] except for lower lip numbness in all selected studies reporting this outcome and for number of lymph node dissected outcome for Chi et al. [26], Ma el al. [20] Teng el al. [19].

On the other hand, TOETSMVA was statistically better than COT in terms of (drainage volume, pain (VAS score), Cosmetic effect, and satisfaction score) but not statistically better than COT in terms of (operation time, hospital stay, number of lymph node resected, intraoperative blood loss. Our results were consistent with the results of Li et al. [24], Chen et al. [28], Zhi-qiang et al. [27] except for drainage volume and pain (VAS score) in Chen et al. [28], Zhi-qiang et al. [27].

### Strengths and limitations

Our study has several strength points: This is the first meta-analysis to evaluate operative outcomes and complications of TOETSMVA compared with other techniques. We reviewed strictly the bibliography of the included studies to include all possible available studies on this topic. We used all the possible outcomes available in the analysis. The study has some limitations that should be considered; a small number of studies with short duration and non-multicentral were included. There are only two studies of seven are RCT and the others are observational studies. The included studies did not have data separately on the type of COT (lobectomy or hemithyroidectomy or total), also on the preservation of the parathyroid gland. Some of the secondary outcomes were reported only by two studies which may limit the generalizability of these outcomes. Also, the cosmetic effect of TOETSMVA in comparison to TOETVA was reported by one study, so the analysis was not feasible. So further studies with larger sample size, longer follow-up duration, multicentral, and multi-armed trials comparing all these techniques are required to measure all the limitations to develop an evidence-based medicine about this potential technique.

## Conclusion

Ultimately, this meta-analysis suggests that the TOETSMVA showed a significant improvement compared to the TOETVA in operation time, hospital stay, number of resected lymph nodes, mandibular numbness, and return to normal diet but did not show a difference in drainage volume, showed a potential effect and providing valuable insight into using this novel approach. However, TOETSMVA is disappointing in comparison to COT except in cosmetic effect, satisfaction score, drainage volume, and pain VAS score. Further RCTs with larger sample size, multicentral, and longer follow-up duration are necessary to evaluate the limitations of TOETSMVA.

### Electronic supplementary material

Below is the link to the electronic supplementary material.

Supplementary Material 1

Supplementary Material 2

## Data Availability

Data is provided within the manuscript or supplementary information files.
