# Supplementary material for: Transoral endoscopic thyroidectomy submental vestibular approach for early-stage papillary thyroid carcinoma: a systematic review and meta-analysis
Source: Langenbecks Arch Surg. 2024 Jul 4;409(1):204. doi: 10.1007/s00423-024-03377-x (PMC11224072; doi:10.1007/s00423-024-03377-x)
Supplement: Supplementary file 1 — Supplementary Material 1 [file 423_2024_3377_MOESM1_ESM.pdf]

Contents

Title....................2

Running Title....................2

Authors....................2

Affiliations....................2

Corresponding author....................2

ESM. 1....................3

ESM. 2....................3

ESM. 3....................3

ESM. 4....................4

ESM. 5....................4

ESM. 6....................4

ESM. 7....................5

ESM. 8....................5

ESM. 9....................6

Title.

Transoral Endoscopic Thyroidectomy Submental Vestibular Approach for Early-Stage Papillary Thyroid Carcinoma: A Systematic Review and Meta-Analysis

Running Title.

TOETSMVA for Papillary Thyroid Carcinoma

Authors.

- Mahmoud Diaa Hindawi
- Ahmed Hamdy G. Ali
- Ruaa Mustafa Qafesha
- Wesam Soliman
- Haitham Salem
- Eslam Bali
- Amr Elrosasy

Affiliations.

Faculty of Medicine, Al-Azhar University, Cairo, Egypt.

Faculty of Medicine, Ogarev Mordovia State University, Saransk, Russia.

Medical Research Group of Egypt, Negida Academy, Arlington, Massachusetts, USA.

Faculty of Medicine, Al-Quds University, Jerusalem, Palestine.

Faculty of Medicine, Assiut University, Assiut, Egypt.

Faculty of medicine, Ain shams University, Cairo, Egypt.

Faculty of Medicine Cairo University, Cairo, Egypt.

Keywords:

Papillary thyroid carcinoma; endoscopic thyroidectomy; transoral endoscopic thyroidectomy submental vestibular approach; transoral endoscopic thyroidectomy vestibular approach; conventional open thyroidectomy.

Corresponding author

Amr Elrosasy

Faculty of Medicine Cairo University, Cairo, Egypt.

Email: 10912022103193@stud.cu.edu.eg

ORCID: https://orcid.org/0000-0002-5592-3908

ESM. 1

(Endoscopic thyroidectomy OR Endoscopic thyroid surgery OR minimally invasive thyroid surgery OR Submental OR Submental-transoral OR Transoral and submental endoscopic thyroidectomy OR TOETSMVA OR Transoral endoscopic thyroidectomy via vestibular approach OR TOETVA) AND (Thyroidectomy OR Total thyroidectomy OR TT OR Hemithyroidectomy OR subtotal thyroidectomy OR Lobectomy) AND (Papillary thyroid carcinoma OR Carcinoma OR Papillary)

ESM. 2

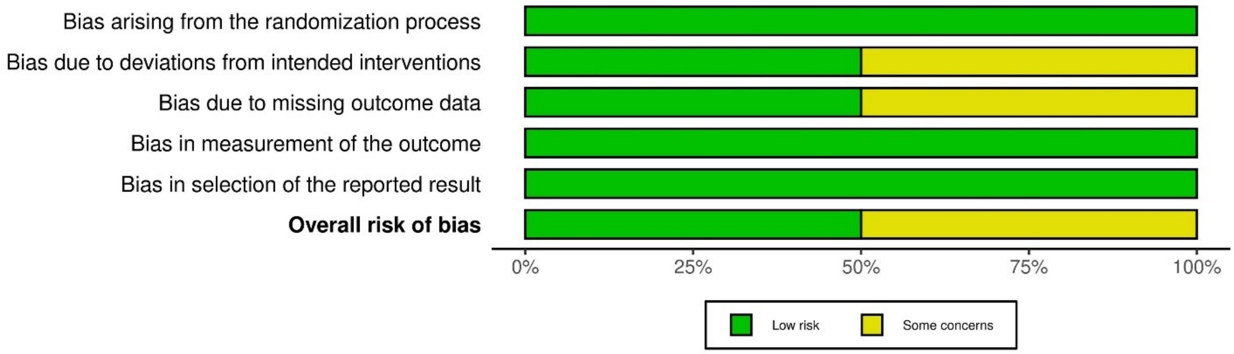

ESM. 2. Risk of bias graph for randomized controlled trials using ROB2

ESM. 3

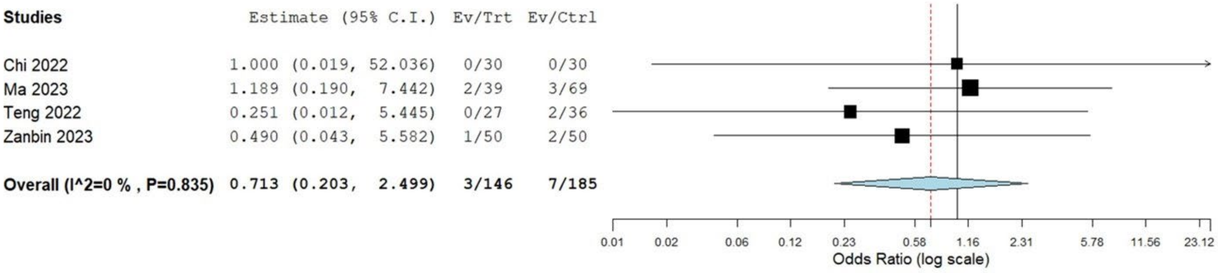

ESM. 3. Meta-analysis forest plot using Open Meta-Analyst for drinking or cough incidence

ESM. 4.

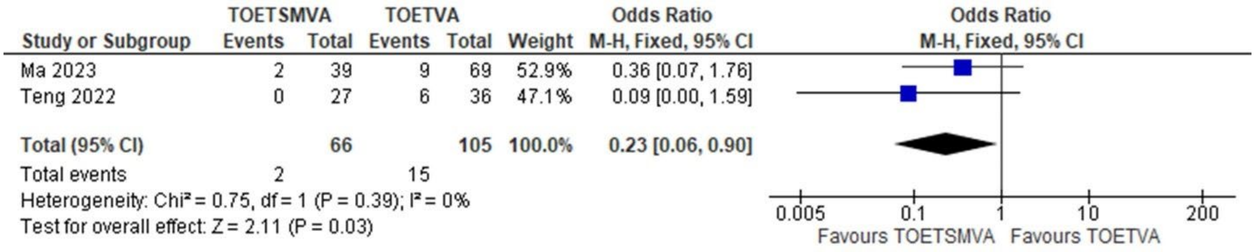

ESM. 4. Meta-analysis forest plots containing mandibular numbness.

ESM. 5.

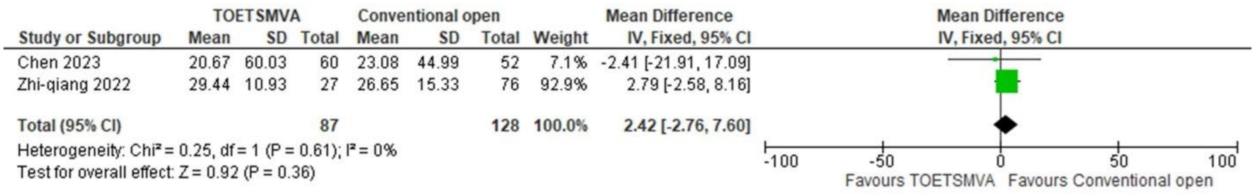

ESM. 5. Meta-analysis forest plots containing intraoperative blood loss.

ESM. 6.

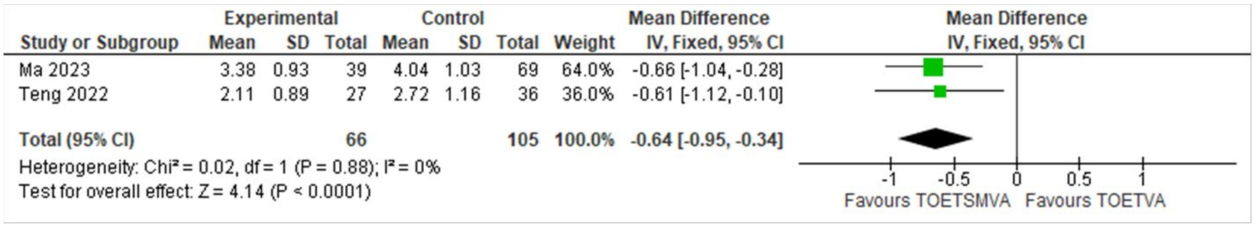

ESM. 6. Meta-analysis forest plots containing return to normal diet.

ESM. 7.

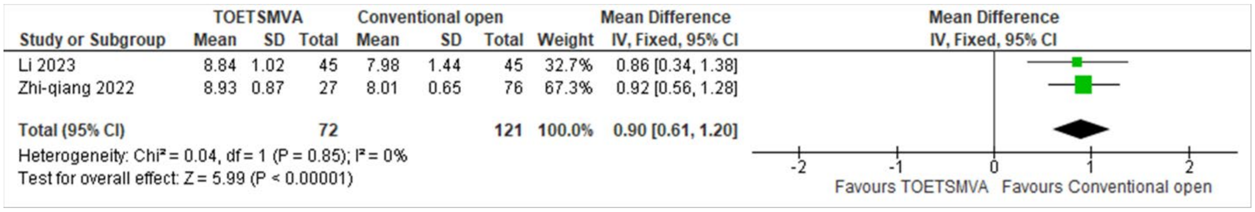

ESM. 7. Meta-analysis forest plots containing satisfaction score.

ESM. 8.

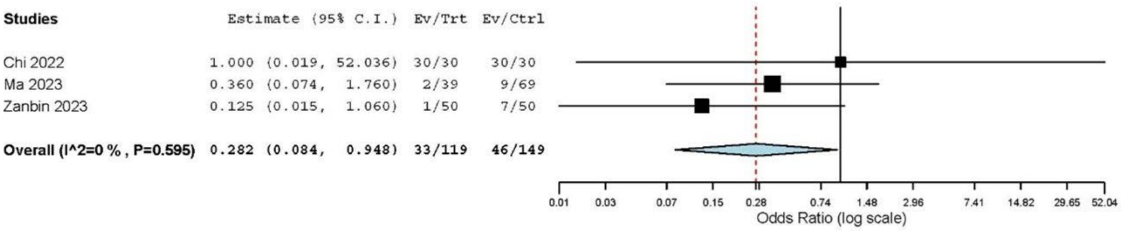

ESM. 8. Meta-analysis forest plot using Open Meta-Analyst for lower lip numbness.

ESM. 9.

Certainty assessment						Summary of findings				
						Study event rates (%)			Anticipated absolute effects	
Participants (studies) Follow-up	Risk of bias	Inconsistency	Indirectness	Imprecision	Overall certainty of evidence	With [comparison]	With [intervention]	Relative effect (95% CI)	Risk with [comparison]	Risk difference with [intervention]
**Drinking or coughing incidence**										
331 (1 RCT, 3 observational)	Not serious	Not Serious	Not serious	Serious[b]	⊕⊕◯◯ Low	7/185 (3.78%)	3/146 (2.05%)	RR 0.713 (0.203 to 2.499)	38 per 1000	21 per 1000
**Mandibular numbness**										
171 (2 observational)	Not serious	Not serious	Serious	Serious[b]	⊕⊕◯◯ Low	15/105 (14.2%)	2/66 (3.03%)	RR 0.23 (0.06 to 0.9)	143 per 1000	30 per 1000
**Lower lip numbness**										
268 (1 RCT, 2 observational)	Not serious	Not serious	Serious	Serious[b]	⊕◯◯◯ Very low	33/119 (27.7%)	46/149 (30.8%)	RR 0.282 (0.084 to 0.948)	277 per 1000	309 per 1000
**Temporary hoarseness**										
331 (1 RCT, 3 observational)	Not serious	Not serious	Not serious	Serious[b]	⊕⊕◯◯ Low	9/185 (4.86%)	8/146 (5.4%)	RR 1.12 (0.45 to 2.75)	49 per 1000	55 per 1000
**Recurrent laryngeal nerve injury**										
361 (1 RCT, 3 observational)	Not serious	Not serious	Not serious	Very serious	⊕◯◯◯ Very low	6/141 4.2%)	10/220 (4.5%)	RR 1.7 (0.33 to 8.74) for subgroup 1 and RR 0.69 (0.18 to 2.67) for subgroup 2	41 per 1000	45 per 1000

Table 3: GRADE evidence profile.

CI: confidence interval; RR: risk ratio.

Explanations

a. I-square test > 70%.
b. A few numbers of events (>300 events).
c. Confidence interval does not exclude the risk of appreciable benefit/harm, and few number of events (< 300 event).
d. Confidence interval does not exclude the risk of appreciable benefit/harm.
